# Supplementary material for: GTPase-activating protein Rasal1 associates with ZAP-70 of the TCR and negatively regulates T-cell tumor immunity
Source: Nat Commun. 2019 Oct 22;10:4804. doi: 10.1038/s41467-019-12544-4 (PMC6805919; doi:10.1038/s41467-019-12544-4)
Supplement: Supplementary file 1 — Supplementary Information [file 41467_2019_12544_MOESM1_ESM.pdf]

Supplementary Figures

Thaker et al (2019)

SFigure 1

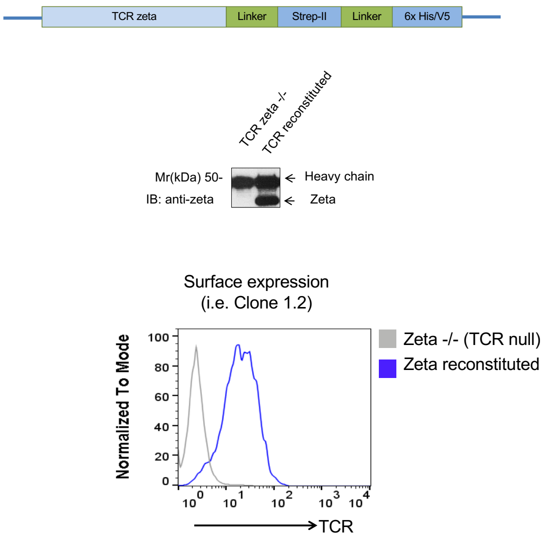

SFigure 1: Tandem affinity purification (TAP) from reconstituted TCR-null zeta$^{-/-}$ 3A9 cells. Upper panel: structure of TAP zeta chain construct with tandem Step-II and His/V5 tags for purification. Middle panel: presence of the TAP zeta chain in constituted TCR-null zeta$^{-/-}$ 3A9 cells. Lower panel: flow cytometry showing the surface expression of the TCR complex on TCR-null zeta$^{-/-}$ 3A9 cells transfected to express TAP zeta complex.

SFigure 2

EXPERIMENT 1

Protein Accession	Protein Description	Protein Mascot Score	Protein Expect	Protein Mass	Protein Matches	Peptide Rank	Peptide sequence
sp|P24161-1|CD3Z_MOUSE	Isoform CD-3-zeta of T-cell surface glycoprotein CD3 zeta chain OS=Mus musculus GN=Cd247	675	1.60E-63	18740	45	1	EEYDVLEK
sp|P24161-1|CD3Z_MOUSE	Isoform CD-3-zeta of T-cell surface glycoprotein CD3 zeta chain OS=Mus musculus GN=Cd247	508	8.80E-47	18740	25	1	REEYDVLEK
tr|E9Q557|E9Q557_MOUSE	Uncharacterized protein OS=Mus musculus GN=Dsp	489	7.00E-45	335158	23	1	AQIDNLTR
tr|E9Q1Z0|E9Q1Z0_MOUSE	Uncharacterized protein OS=Mus musculus GN=4732456N10Rik[]RIKEN cDNA 4732456N10 gene	446	1.30E-40	58587	33	1	FASFIDK
tr|E9Q386|E9Q386_MOUSE	Uncharacterized protein OS=Mus musculus GN=Cd3g	336	1.30E-29	20725	16	1	NLVQVDGSR
sp|P04235|CD3D_MOUSE	T-cell surface glycoprotein CD3 delta chain OS=Mus musculus GN=Cd3d	220	5.40E-18	19305	13	1	LGGNWPR
tr|A0N8J3|A0N8J3_MOUSE	Tcell receptor chain (Fragment) OS=Mus musculus GN=Trbc2	131	4.30E-09	32068	11	1	VSLFEPSK
sp|P22646|CD3E_MOUSE	T-cell surface glycoprotein CD3 epsilon chain OS=Mus musculus GN=Cd3e	131	4.60E-09	21779	13	1	DLYSGLNQR
tr|A2NTU7|A2NTU7_MOUSE	Cytotoxic Tcell receptor OS=Mus musculus GN=Trac	124	2.10E-08	30063	6	1	TMESGTFITDK
tr|A2NTY6|A2NTY6_MOUSE	Beta-chain (Fragment) OS=Mus musculus	107	1.00E-06	15916	8	1	GDIPDGYK
tr|E9Q4P0|E9Q4P0_MOUSE	Uncharacterized protein OS=Mus musculus GN=2810422J05Rik	71	0.0046	22277	3	1	TITLEVEPSDTIENVK
tr|B2RQC5|B2RQC5_MOUSE	Tyrosine-protein kinase receptor OS=Mus musculus GN=Insrr	63	0.03	147291	5	1	SEVTELR
**sp|Q9Z268|RASL1_MOUSE**	**RasGAP-activating-like protein 1 OS=Mus musculus GN=Rasal1**	**61**	**0.03**	**90285**	**4**	**1**	**EAPGTTSPLR**
sp|A2A4P0|DHX8_MOUSE	ATP-dependent RNA helicase DHX8 OS=Mus musculus GN=Dhx8	57	0.11	143337	3	1	VAAMSVAK
tr|A2MZG6|A2MZG6_MOUSE	CD3 theta OS=Mus musculus GN=Cd247	56	0.14	21549	2	1	NPQEGVYNALQK
tr|D3Z4W5|D3Z4W5_MOUSE	Uncharacterized protein OS=Mus musculus GN=1700074P13Rik	49	0.75	20422	6	1	NDLMLIK
tr|D3Z4W5|D3Z4W5_MOUSE	Uncharacterized protein OS=Mus musculus GN=1700074P13Rik	47	1.1	20422	6	1	NDLMLIK
tr|E9QL28|E9QL28_MOUSE	Uncharacterized protein OS=Mus musculus GN=Igfn1	47	1.1	140949	3	1	LTVIEDLR
tr|Q9CPN9|Q9CPN9_MOUSE	RIKEN cDNA 2210010C04, isoform CRA_b OS=Mus musculus GN=2210010C04Rik	46	1.4	27089	1	1	YVNWIQQTIAAN
tr|D3YX85|D3YX85_MOUSE	Uncharacterized protein OS=Mus musculus GN=6530401G17Rik	43	2.6	107593	3	1	EIISEVQR

EXPERIMENT 2

Protein Accession	Protein Description	Protein Mascot Score	Protein Expect	Protein Mass	Protein Matches	Peptide Rank	Peptide sequence
sp|A2AFR3-1|FRPD4_MOUSE	Isoform 1 of FERM and PDZ domain-containing protein 4 OS=Mus musculus GN=Frmpd4	44	2.3	146084	3	1	KAQESPR
sp|P01739|TVA2_MOUSE	T-cell receptor alpha chain V region 2B4 OS=Mus musculus	146	1.30E-10	14773	9	1	ALMSIFSNGEK
sp|P04235|CD3D_MOUSE	T-cell surface glycoprotein CD3 delta chain OS=Mus musculus GN=Cd3d	288	8.60E-25	19305	25	1	LGGNWPR
sp|P10126|EF1A1_MOUSE	Elongation factor 1-alpha 1 OS=Mus musculus GN=Eef1a1	68	0.0081	50424	5	1	LPLQDVYK
sp|P17897|LYZ1_MOUSE	Lysozyme C-1 OS=Mus musculus GN=Lyz1	53	0.29	17240	1	1	STDYGIFQINSR
sp|P20029|GRP78_MOUSE	78 kDa glucose-regulated protein OS=Mus musculus GN=Hspa5	508	8.30E-47	72492	22	2	VLEDSDLK
sp|P22646|CD3E_MOUSE	T-cell surface glycoprotein CD3 epsilon chain OS=Mus musculus GN=Cd3e	460	6.10E-42	21779	67	1	GTGAGSRPR
sp|P24161-1|CD3Z_MOUSE	Isoform CD-3-zeta of T-cell surface glycoprotein CD3 zeta chain OS=Mus musculus GN=Cd247	652	3.60E-61	18740	110	1	ARDPEMGGK
sp|P62897|CYC_MOUSE	Cytochrome c, somatic OS=Mus musculus GN=Cycs	47	1.1	11712	3	1	ADLIAYLK
sp|P63017|HSP7C_MOUSE	Heat shock cognate 71 kDa protein OS=Mus musculus GN=Hspa8	500	5.10E-46	71055	23	1	NTTIPTK
sp|Q5F2E7-1|NUFP2_MOUSE	Isoform 1 of Nuclear fragile X mental retardation-interacting protein 2 OS=Mus musculus GN=N	715	1.60E-67	75668	27	1	SGETQAVDK
sp|Q61171|PRDX2_MOUSE	Peroxiredoxin-2 OS=Mus musculus GN=Prdx2	41	4	21936	3	1	QITVNDLPVGR
sp|Q91VR5|DDX1_MOUSE	ATP-dependent RNA helicase DDX1 OS=Mus musculus GN=Ddx1	187	1.10E-14	83474	12	1	VPVDEFDGK
sp|Q99LF4|RTCB_MOUSE	tRNA-splicing ligase RtcB homolog OS=Mus musculus GN=D10Wsu52e	62	0.032	55727	3	1	TNLDESDVQPVK
sp|Q9CQ48|NUDC2_MOUSE	NudC domain-containing protein 2 OS=Mus musculus GN=Nudcd2	44	2.1	17820	2	1	MSAPFEER
sp|Q9CQB2|F195A_MOUSE	Protein FAM195A OS=Mus musculus GN=Fam195a	103	2.90E-06	17974	5	1	TGPTQQQVESR
**sp|Q9Z268|RASL1_MOUSE**	**RasGAP-activating-like protein 1 OS=Mus musculus GN=Rasal1**	**49**	**0.03**	**90285**	**4**	**1**	**RDLAPRDISGTS**
tr|A0N8J3|A0N8J3_MOUSE	Tcell receptor chain (Fragment) OS=Mus musculus GN=Trbc2	55	0.19	32068	3	1	EVHSGVSTDPQAYK
tr|A2A9Z0|A2A9Z0_MOUSE	Dystrophin, muscular dystrophy OS=Mus musculus GN=Dmd	52	0.35	427676	3	1	EEIKIK
tr|A2AI63|A2AI63_MOUSE	Hephaestin OS=Mus musculus GN=Heph	43	2.8	130496	3	1	GLTVYQGER

* protein hits from control samples (TCR deficient 3A9 T cells) and common contaminats have been exlcuded.
* a representative peptide from each hit is shown

SFigure 2: Rasal1 peptides are detected by TCR complex by tandem affinity purification (TAP) and MS/MS analysis. Peptides detected by MS/MS from 2 separate experiments.

SFigure 3

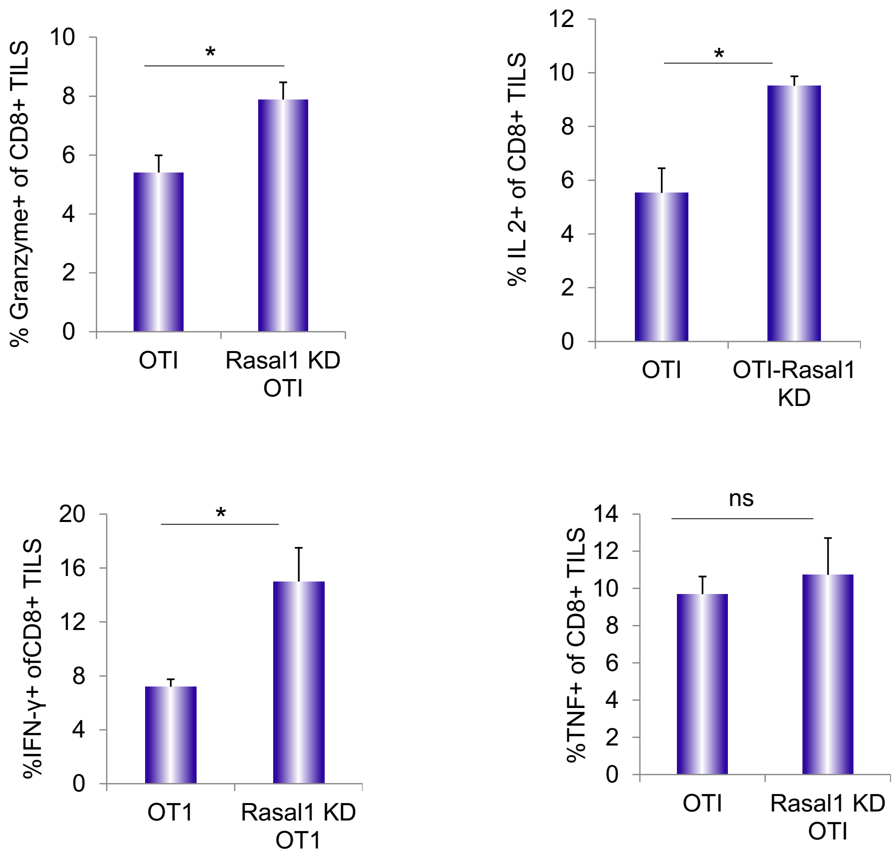

SFigure 3: MFI expression values for granzyme B and cytokines on scrambled siRNA or Rasal1 KD siRNA T-cells**.** Histogram showing the % of CD8 TILs expressing granzyme, Interleukin 2, IFN-γ1 and TNFα. T-cells were transfected with scrambled siRNA or Rasal1 KD siRNA T-cells followed by removal of TILs from EL4 tumors as described in the text.

Fig. 1B

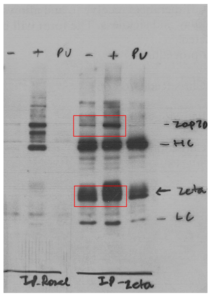

Fig. 1D

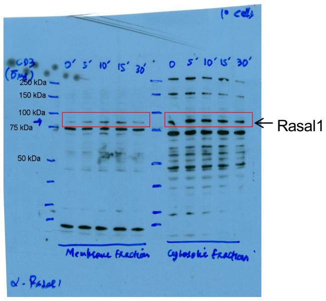

Fig. 2B

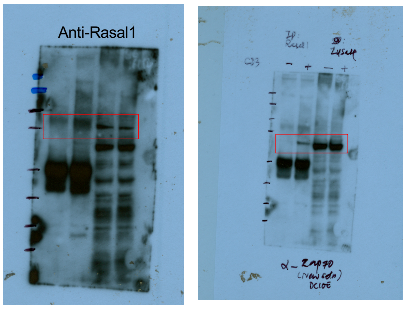

Fig. 2C

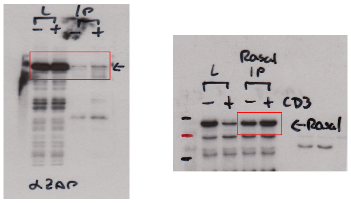

SFigure 4: Uncropped versions of Figures 1-2

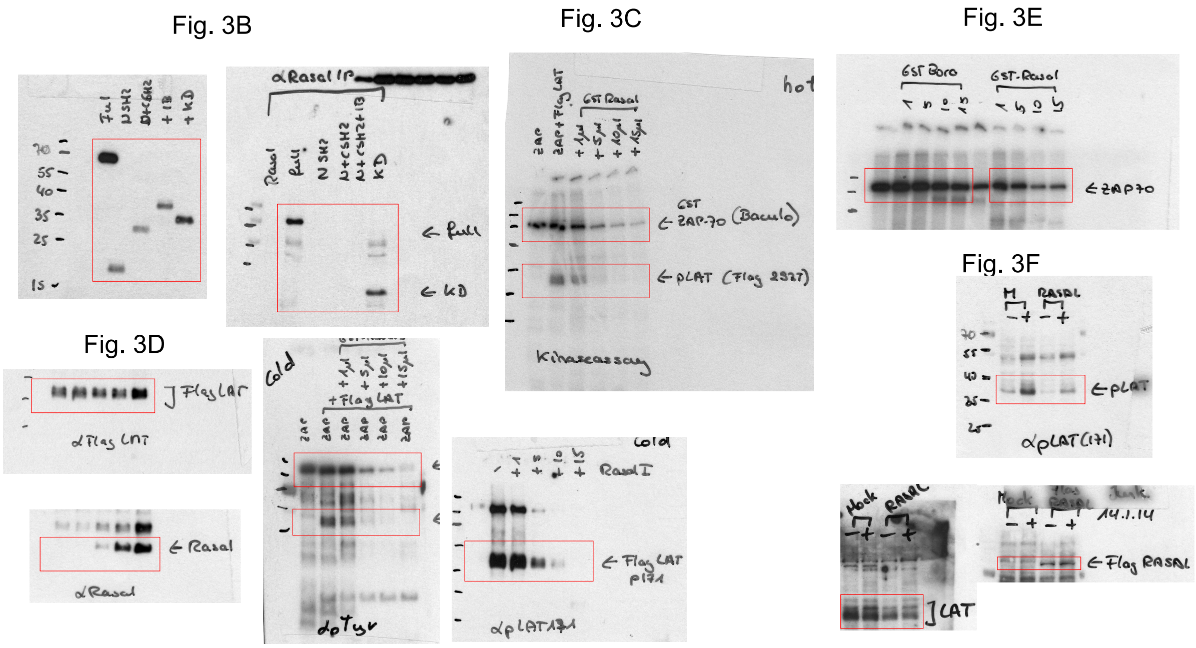

SFigure 4: Uncropped versions of Figure 3

Fig. 4A (top)

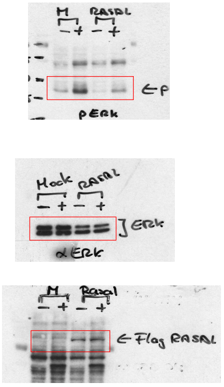

Fig. 4A (bottom)

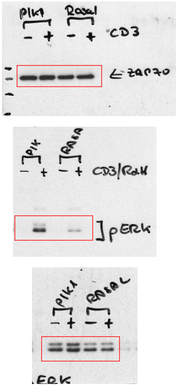

Fig. 4A inset

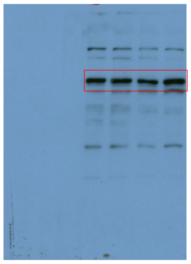

Fig. 4C

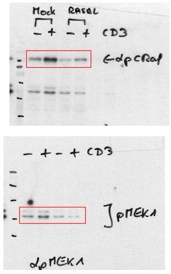

Fig. 5A uncropped

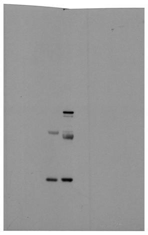

Fig. 5D uncropped

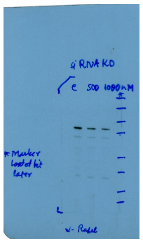

SFigure 4: Uncropped versions of figures 4-5
